# Hsa_circ_0020095 modulates chemoresistance of CRC in a PDO model

**DOI:** 10.3389/fmed.2025.1556611

**Published:** 2025-05-27

**Authors:** Xinyu Li, Tao Li, Yan Zhao, Junqi Shan, Yang Gao, Changchun Zhou, Yanlai Sun

**Affiliations:** ^1^School of Clinical Medicine, Shandong Second Medical University, Weifang, Shandong, China; ^2^Department of Surgical Oncology, Shandong Cancer Hospital and Institute, Shandong First Medical University and Shandong Academy of Medical Sciences, Jinan, Shandong, China; ^3^Graduate School of Shandong First Medical University, Shandong Academy of Medical Sciences, Jinan, Shandong, China; ^4^Shandong Provincial Key Laboratory of Precision Oncology, Shandong Cancer Hospital and Institute, Shandong First Medical University and Shandong Academy of Medical Sciences, Jinan, Shandong, China; ^5^School of Clinical Medicine, Jining Medical University, Jining, China

**Keywords:** Hsa_circ_0020095, CRC, organoids, chemoresistance, POD abbreviation

## Abstract

**Background:**

Colorectal cancer (CRC) is the third most common malignant tumor type all over the world with high mortality. Chemoresistance of CRC leads to treatment failure and disease aggravation. We previously identified Hsa_circ_0020095 as a novel oncogene to promote progression and cisplatin-resistance in colon cancers by modulating the miR-487a-3p/SOX9 axis.

**Methods:**

Patient-derived organoids (PDOs) were generated from CRC patients and validated by H&E staining, immunohistochemistry (IHC), and whole exome sequencing (WES). Hsa_circ_0020095 was knocked down in PDOs by shRNA and the inhibition of hsa_circ_0020095 was determined using RT-qPCR. The RNA samples analyzed separately, and then pooled together for KEGG and GO analyses. The effects of knocking down hsa_circ_0020095 on drug-resistance of PDOs were examined using CellTiter-Glo^®^3D Cell viability assay. Finally, the underlying mechanism was explored by transcriptomic sequencing and subsequent bioinformatics analyses.

**Results:**

Five organoid lines were successfully established from CRC patients using surgically resected tumor samples. PDOs resembled their parental tumor tissues in morphology, histopathology, and genetic alterations. Silencing of circ_0020095 resulted in remarkable inhibition of hsa_circ_0020095 in PDOs and reversed the resistance of PDOs to 5-FU and oxaliplatin. Mechanistically, hsa_circ_0020095 may function by modulating key pathways and biological functions involved in pathophysiological processes in CRC.

**Conclusion:**

Hsa_circ_0020095 modulates chemoresistance of CRC, which could potentially be explored as a therapeutic target for CRC treatment.

## Introduction

Colorectal cancer (CRC) ranks the third most common malignancy worldwide, exhibiting high incidence and mortality (1). In China alone, there were 870, 000 and 767, 000 new cases and deaths from CRC in 2022, respectively, while the numbers are still raising steadily (2). Although surgical resection and systemic therapies are essential to treat CRC, the overall 5 years survival rate of patients remains unsatisfactory (3). 5-fluorouracil (5-FU) and oxaliplatin are cornerstone agents for the systemic treatments of CRC; however, drug resistance, whether inherent or acquired, limits the clinical benefits (4). Therefore, it is imperative to increase the sensitivity of CRC cells to 5-FU/Oxaliplatin for more effective treatment.

Circular RNAs (circRNAs) are a large class of non-coding RNAs in eukaryotes that are produced by backsplicing, a non-canonical splicing event. They were originally considered as “junk” generated by aberrant splicing events, however, recent researches demonstrated their comprehensive existence and complicated biological functions by means of miRNA or protein sponges, which are still largely unexplored (5). CircRNAs can interact with miRNAs, mRNAs or RNA-binding proteins, thus activating or repressing expression of target genes (6). Many of the target gene products are cellular components that play key roles in cancer-related signaling pathways, underling the contribution of cirRNAs in cancers (7). For example, ciRS-7 activates the oncogenes EGFR and RAF1, thereby activating the EGFR/RAF1/MAPK pathway and promoting progression of CRCs, by sponging and suppressing miR-7 activity (8); while circHAS2 activates CCNE2 by sponging miR-1244 to enhance cell proliferation and sensitivity of CRC to anlotinib, a multi-target TKI used to treat cancers (9). In our previous work, we have identified hsa_circ_0020095 as a novel oncogene to promote progression and cisplatin-resistance in colon cancers by modulating the miR-487a-3p/SOX9 axis (10). Therefore, hsa_circ_0020095 could potentially be explored as a biomarker and therapeutic target for CRC. However, it remains unclear whether hsa_circ_0020095 could also reverse the unresponsiveness of refractory CRCs toward 5-FU and oxaliplatin. Besides, much of the evidence of cirRNAs came from studies using cell line assays (11), which may not reflect the high heterogeneity of tumors’ responses to systemic therapy observed clinically.

In the current study, we will further explore the potential roles of hsa_circ_0020095 in modulating drug resistance of CRCs and its underlying mechanism, using patient-derived organoids (PDOs), micro-organs that are derived from human tissue while resemble the tumor in both the appearance and molecular and genetic characteristics.

## Materials and methods

### Clinical sample collection

This study was approved by the Ethical Committee of Shandong Cancer Hospital (SDTHEC2023006021), and informed consent was obtained from all participants. Surgically untreated resected colorectal cancer tissues were transported to the laboratory at 4°C for subsequent processing.

### Tumor cell isolation and preprocessing

Following an optimized tissue digestion protocol (12), tumor samples were washed three times with pre-chilled buffer containing Advanced DMEM/F12 medium (Invitrogen) and 2% penicillin/streptomycin (Gibco), 5 min each, and mechanically minced into fragments smaller than 1 mm^3^. A composite enzyme digestion system was used for digestion containing 500 U/mL collagenase IV (Sigma-aldrich), 1.5 mg/mL collagenase II (Solarbio), 20 mg/mL hyaluronidase (Solarbio), 0.1 mg/mL dispase II (Sigma), 10 μM ROCK inhibitor Y-27632 (Sigma-aldrich), and 1% FBS in DMEM (Lonza) at 37°C in a constant-temperature shaker for 30 min. The digested product was sequentially filtered through 100 and 70 μm cell strainers to obtain a single-cell suspension.

### Organoid culture

A total of 1.5 × 106 CRC cells were rinsed with DMEM/F12 (Invitrogen), then mixed with Matrigel (20 μL/well) and seeded in a 48-well plate. One-CULTar™ intestinal tumor-specific medium was added, and the medium was replaced every 3–4 days. When organoids reached 70% confluency, TrypLE™ Express (GIBCO) was used to dissolve the Matrigel, and mechanical pipetting was performed to obtain organoid fragments. After washing with PBS to remove Matrigel, the fragments were re-embedded in Matrigel for subculture. Morphological changes of the organoids were recorded throughout the process using an inverted microscope (Olympus, Tokyo, Japan).

The composition of the culture medium was as follows (13): advanced DMEM/F12 (ThermoFisher) with 1% HEPES buffer (ThermoFisher), 1% Glutamax (ThermoFisher) and 1% Penicillin/Streptomycin (P/S, ThermoFisher), 20% R-spondin conditioned medium, 10% Noggin conditioned medium, 1X B27 (ThermoFisher), 1.25 mM n-Acetyl Cysteine (Sigma-Aldrich), 10 mM Nicotinamide (Sigma-Aldrich), 50 ng/mL EGF (Peprotech), 500 nM A83-01 (Tocris), 10 mM SB202190 (Gentaur), and the organoids cultured for the first 2 days with 10 μM Y-27632 (Sigma-aldrich) for maintenance.

### Cell transfection

The siRNAs targeting hsa_circ_0020095 and the control siRNAs were desigened and synthesized by HanBio (Shanghai, China). The target gene sequences were as follows: siRNA#1: AGTGTCCAGCATCAAACACTT, siRNA#2: CAGTGTCCAGCATCAAACACT. Organoids were transfected with KD or NC plasmids according to the manufacturer’s instructions when cells reached 70% confluence. Transfection efficiency was determined by RT-qPCR.

### H&E and immunohistochemistry (IHC)

Organoids and paired tumor tissues were fixed with 4% paraformaldehyde and embedded in paraffin for sectioning. After that, deparaffinized sections of CRC tissues and organoid were subjected to H&E and IHC staining. The following antibodies were used: (1) Anti-CDX-2 antibody (1:400, ab76541, Abcam), (2) Anti-CK7 antibody (1:100, ab181598, Abcam), (3) Anti-Ki67 antibody (1:250, ab16667, Abcam), (4) Anti-CK20 antibody (1:250, ab76126, Abcam); (5), Anti-Villin antibody (1:100, ab130751, Abcam). Positive staining cells in five random fields of each section were visualized using an inverted microscope (Olympus, Tokyo, Japan) and quantitatively analyzed using Image-Pro Plus 6.0 software (Media Cybernetics Inc., Bethesda, United States).

### Whole exome sequencing

DNA was extracted from organoids and resected frozen tumor using the QIAamp DNA Mini Kit (QIAGEN), according to the manufacturer’s instructions. The DNA libraries were prepared using the SureSelect Human All Exon V6 Kit (Agilent Technologies), and samples were sequenced using a NovaSeq 6000 (Illumina). Sequence alignment and mutation calling were performed as follows: to remove adaptors and filter low quality reads of raw fastq datas using Fastp (v0.23.2) software. And then Sequence alignment against the human reference genome (hg38) using BWA-MEM (v0.7.17). Somatic single-nucleotide variant (SNVs) and indels in the tumor and paired organoids were called by GATK (v4.0.5.1).

### RT-qPCR

TRIzol^®^ reagent (Takara, Tokyo, Japan) was used to isolate total RNA. PrimeScript RT kit (ELK bioscience, Wuhan, China) was used to reversely transcribe total RNA into cDNA. After that, RT-qPCR was performed using the SYBR premix Ex Taq II kit (ELK bioscience). Real-Time qPCRs were performed in triplicate. The protocol of amplification was as follows: 2 min at 94°C, followed by 35 cycles (30 s at 94°C and 45 s at 55°C). The primer sequences were as follows: hsa_circ_0020095 forward, 5′-GCTTATTGGAATGCACCACA-3′ and reverse, 5′-GTTTCTGGAACAAGCCAAGTG-3′; GAPDH forward, 5′-TGTTCGTCATGGGTGTGAAC-3′ and reverse, 5′-AT GGCATGGACTGTGGTCAT-3′; ACTL8 forward, 5′-CTCTCC GCCTATCTCCTCAAG-3′ and reverse, 5′-TAGGTGTTGCTCT CATCCAAGG-3′; CATSPERZ forward, 5′-CCTGATGGAACT CATGGAGAAT-3′ and reverse, 5′-ATTCACGTACAGCCTC TTGCTC-3′; SLC1A3 forward, 5′-AATGGCGGCGCTAGAT AGTAAG-3′ and reverse, 5′-CTGCAGCTGTCACTCGTACAAT-3′; CR2 forward, 5′-CCCACGCTGTGAACTTTCTACT-3′ and reverse, 5′-CCTTCAAGGTGAAGCCAAACAT-3′; WNT5A forward, 3′-CATCCTCATGAACCTGCACAAC-3′ and reverse, 5′-tGCGCTGTCGTACTTCTCCTTCA-3′; TTYH2 forward, 5′-CCA GATCAGCACAGAGGTGACTT-3′ and reverse, 5′-AAGGCT GGACTCTGAGGAGTTC-3′; TRPM6 forward, 5′-GGTGAAAGA GGAGATCATCTGC-3′ and reverse, 5′-AGTCCAGGTCTTGC TGCTCTTC-3′; IL6R forward, 5′-CCAGAAGTTCTCCTGCCA GTTA-3′; and reverse, 5′-CACGGCAGTGACTGTGATGTT-3′; IFNL2 forward, 5′-GTGACTGGAGCAGTTCCTGTCG-3′ and reverse, 5′-AGTCCTTCAGCAGAAGCGACTC-3′; PWW P3B forward, 5′-GAGCGAGGATACCTGCCTAGA-3′ and reverse, 5′-GAGCGAGGATACCTGCCTAGA-3′; CCDC70 forward, 5′-AAGAGACCGGAACCTTCTTCAG-3′ and reverse, 5′-CCAT TGTTCTCCATCCAGAAG-3′; KLF8 forward, 5′-TGGCTCAAT GCAGGTATTCAA-3′ and reverse, 5′-ATTAAACAGTGCTGGG TTCTCC-3′; NLRP3 forward, 5′-AAGATGGAGTTGCTGTTT GACC-3′ and reverse, 5′-GCTCACCTCTCGACAGTGGATA-3′; TRIM22 forward, 5′-AAGAGAGAACCGCCTGGAAGAT-3′ and reverse, 5′-GAGATCTGAGATGAGCGTGCTG-3′; ZBP1 forward, 5′-CTTCTGGACATGGATGAGCAGT-3′ and reverse, 5′-ATGAT GTTCCCGTGTCCAAT-3′; CXCL10 forward, 5′-GCCATTCTG ATTTGCTGCCT-3′ and reverse, 5′-TCAACACGTGGA CAAAATTGG-3′. The 2^–ΔΔt^ method was used for quantifying the gene expression levels. GAPDH was used as an internal reference.

Total RNA was extracted from organoids using the TransZol Up Plus RNA Kit (TransGen Biotech). RNA sequencing libraries were subsequently prepared with the VAHTS Universal V6 RNA-seq Library Prep Kit (Vazyme). The constructed libraries were sequenced on an Illumina NovaSeq 6000 platform. Raw sequencing data in FASTQ format underwent quality control processing through Fastp software (v0.23.2), followed by alignment to the human reference genome (hg38) using STAR aligner (v2.7.8a). Gene expression quantification was performed with RSEM software (v1.3.3) and normalized to transcripts per million (TPM) values. Differential gene expression analysis identified significantly regulated genes based on predefined thresholds (absolute log2 fold change > 0.585, *p*-value < 0.05), with results visualized through hierarchical clustering heatmaps and volcano plot analysis.

### Drug sensitivity test

Single-cell suspensions of organoids were obtained by enzymatic dissociation and seeded in 96-well plates in 1 μL dome-form Matrigel for drug testing. The medium was replaced by gradient concentrations of 5-FU or oxaliplatin (Selleck) after 48 h. Optical images were captured after drug treatment and organoid viability was evaluated using a CellTiter-Glo3D Cell viability assay (Promega) according to manufacturer’s instruction.

### Transcriptome sequencing

Cellular RNA was extracted for library construction using VAHTS Universal V6 RNA-seq Library Prep Kit (Illumina, United States), and paired-end sequencing was performed on the Illumina NovaSeq 6000 platform (Illumina). The transcriptomic analyses were conducted by OneTar Biomedicine Inc (Shanghai, China) and Genesky Biotechnologies Inc. (Shanghai, China). Adapter sequences were filtered to obtain clean reads. The sequencing results were then aligned to a reference genome GRCh38. Differentially expressed genes (DGEs) were screened as per predefined criteria (|log2FC| > 0.585, *p* < 0.05), and analyzed through heat map and Volcano plots.

### Functional enrichment analysis

For GSEA analyses, gene sets were obtained from MsigDB (H: hallmark gene sets, C2: curated gene sets, C5: ontology gene sets, C6: oncogenic signature gene sets, C7: immunologic signature gene sets, C8: cell type signature gene sets). Expressed genes were ranked by logFC value generated by differential gene analysis using edgeR package (v3.42.4). GSEA analyses were performed by GSEA software (v4.0.3, UC San Diego and Broad Institute, United States). KEGG and GO enrichment analysis were carried out by clusterprofiler package (v4.8.3).

### Differential expression and gene enrichment

The FindAllMarkers function of R package Seurat (version 4.0.4) was used to identify DEGs for each cluster, of which the difference significance was determined by Wilcoxon Rank-Sum test. DEGs in cell state over trajectory were selected for subsequent analysis (average log2 fold change = 0.25, adjusted *p* = 0.05). Genes were enriched by R package clusterProfiler (version 3.18.1) and visualized by ggplot2 package (version 3.3.5).

### Statistical analysis

Data are expressed as the mean ± SD. CellTiter-Glo 3D Cell viability assay and RT-qPCR were repeated in triplicate. In addition, the difference between two groups was analyzed by student’s *t*-test. The comparisons between multiple groups (*n* = 3) were analyzed by one-way analysis of variance and Tukey’s post hoc tests. *P* < 0.05 indicates a statistically significant difference.

## Results

### CRC-PDOs recapitulate the morphology, histology, and genetic alterations of their source tissues

**FIGURE 1:**
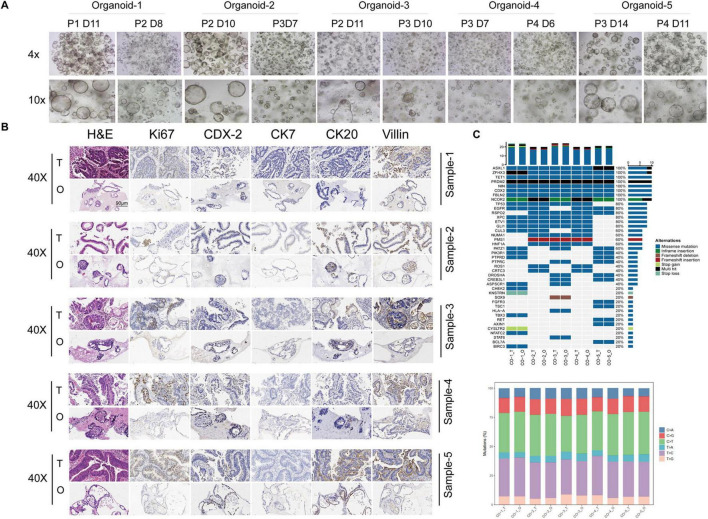
Colorectal cancer (CRC)-patient-derived organoids (PDOs) recapitulate the morphology, histology, and genetic alterations of their source tissues. Five organoid lines were established from CRC patients. Typical morphology of these organoid lines are round or oval shapes with dense edges and transparent cavities. These organoid features were maintained during serial passages **(A)**. Hematoxylin and eeosin (H&E) staining and immunohistochemistry (IHC) of the CRC tissues and their matched organoids. Pathological markers for IHC included Ki67, CK7, Villin, CK20 and CDX-2. Expression of all markers in tumor tissues mentioned above were preserved in their corresponding organoids **(B)**. Whole exome sequencing (WES) was performed on three pairs of organoids and tumor tissues. Genetic alterations commonly observed in CRCs, such as missense mutations **(C**, left**)** and base pair substitutions **(C**, right**)**, are highly consistent between each pair.

Following single cell isolation, PDOs were generated from five CRC patients and serially passaged. Brightfield microscopic imaging, H&E-staining, and IHC analyses were performed to examine the morphological and histological properties of these organoids. Typical organoids exhibited round or oval shapes with dense edges and transparent cavities. These organoid features were maintained during the following serial seedings (Figure 1A). In addition, We compared the expression level of clinical biomarkers between the organoids and the source tissues by IHC staining (Figure 1B), These data show that the CRC-PDOs exhibited the same expression tendencies of clinical biomarkers, and that the CRC-PDOs recapitulated the morphology and histology from their parental CRC biopsies. As drug sensitivity is often associated with genetic alterations in tumors, we next wanted to make certain that these alterations were maintained in CRC-PDOs as their source tissues. We performed WES on these five pairs of organoids and tumor tissues. As illustrated in Figure 1C (left), the most common genetic alterations are missense mutations, and genes unanimously and consistently altered among these organoids and corresponding tissues include ASXL1, ZFHX3, TET1, PRDM2, MIN, CDX2, FBLN2, and NCOR2. Other commonly mutated genes in over 80% of the samples are TP53, EGFR, RSPO2, XPC, ETV1, and GLI1. Besides, base pair substitutions are also highly consistent between each pair (Figure 1C, right).

### Knockdown of hsa_circ_0020095 significantly sensitizes CRC PDOs to 5-FU and oxaliplatin treatments

**FIGURE 2:**
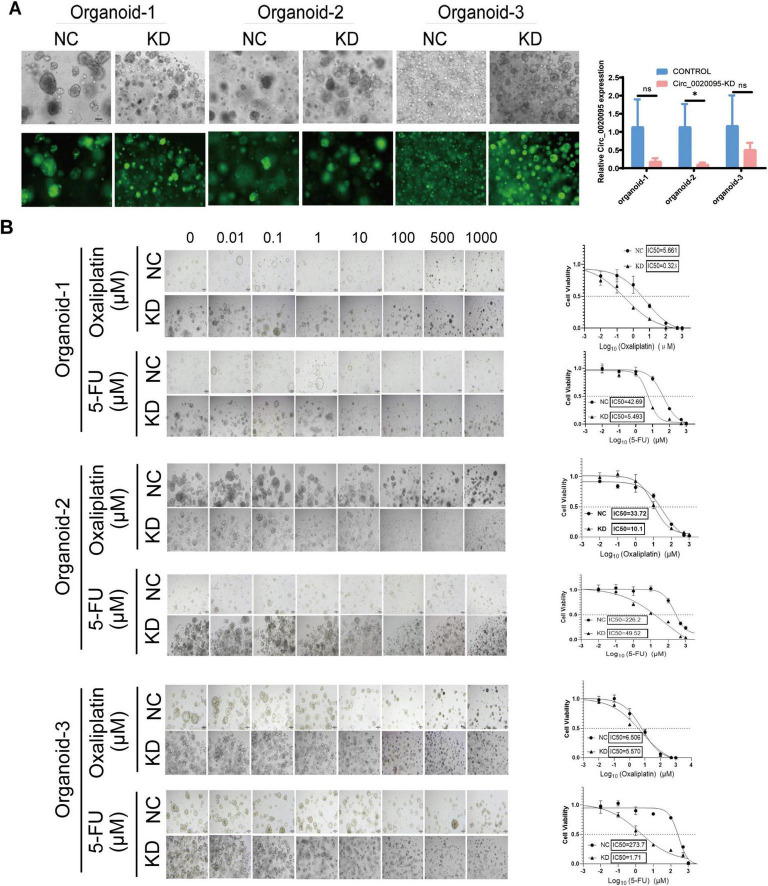
Knockdown of hsa_circ_0020095 significantly sensitizes colorectal cancer (CRC) patient-derived organoids (PDOs) to 5-fluorouracil (5-FU) and oxaliplatin treatments. Three organoid lines were transfected with hsa_circ_0020095 shRNA, and the transfection efficiency was tested by comparing optical and fluorescence images 5 days after the transfection **(A**, left**)**. Knockdown of hsa_circ_0020095 was confirmed by RT-qPCR, the expression level of hsa_circ_0020095 was significantly downregulated in all organoid lines by hsa_circ_0020095 shRNA **(A**, right**)**. A CellTiter-Glo3D Cell viability assay was conducted to determine whether knockdown of hsa_circ_0020095 (KD) in CRC organoids could alter their sensitivity to 5-FU or oxaliplatin, compared to the negative control (NC). and 3 for 5 days. Brightfield optical images were captured before CellTiter- Sequential dilution at a 10-fold gradient of 5-FU or oxaliplatin (from 0 to 1,000 μM) were applied to organoid lines 1, 2, Glo3D Cell viability assay was conducted **(B**, left**)** then dose-response curves were drawn and IC50 values were compared **(B**, right**)**. *P < 0.05.

Next, we wondered whether hsa_circ_0020095 could influence the sensitivity of CRC-PDOs to 5-FU and oxaliplatin. Three organoid lines were transfected with KD and NC, and transfection efficiency was tested by comparing optical and fluorescence images five days after the transfection (Figure 2A, left). Knockdown of hsa_circ_0020095 was confirmed by RT-qPCR as expression level of hsa_circ_0020095 was significantly downregulated in all three organoid lines by hsa_circ_0020095 shRNA, although more remarkable in organoid samples 1 and 2 (Figure 2A, right).

**FIGURE 3:**
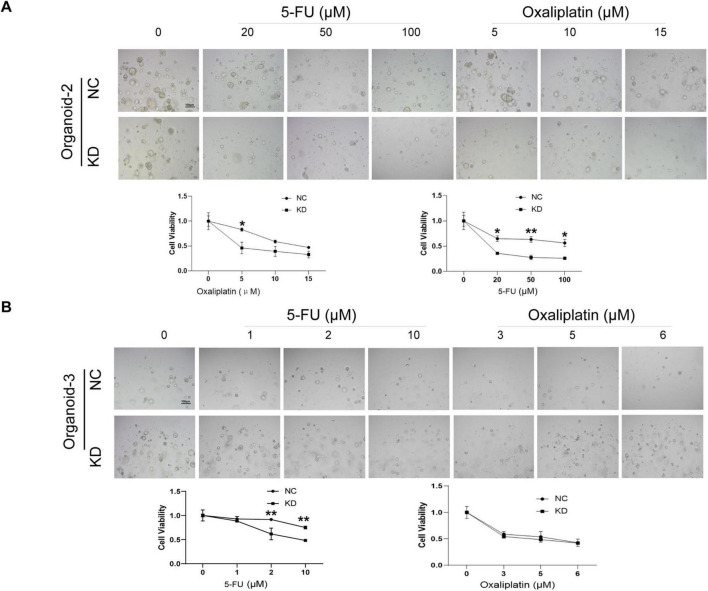
Validation of the sensitivity-enhancing roles of hsa_circ_0020095 in colorectal cancer (CRC) patient-derived organoids (PDOs) to 5-fluorouracil (5-FU) and oxaliplatin. A CellTiter-Glo3D Cell viability assay was conducted after organoids were transfected with hsa_circ_0020095 (KD) or NC and treated with 5-FU or oxaliplatin, at concentrations around their IC_50_ values using Organoid-lines 1 **(A)** and 2 **(B)**. Brightfield optical images were captured before CellTiter-Glo3D Cell viability assay was conducted **(A,B**, upper**)** then dose-response curves were drawn and IC_50_ values were compared **(A,B**, lower**)**. **P < 0.01 compared to negative control (NC).

Afterwards, a CellTiter-Glo3D Cell viability assay was conducted to determine whether knockdown of hsa_circ_0020095 in these organoids could alter their sensitivity to anti-tumor therapeutics such as oxaliplatin and 5-FU. After sequential dilution at a 10-fold gradient, oxaliplatin (from 0 to 1,000 μM) and 5-FU (from 0 to 1,000 μM) were applied to organoid lines 1, 2, and 3 for 5 days. After that, brightfield optical images were captured before CellTiter-Glo3D Cell viability assay was conducted. Knockdown of hsa_circ_0020095 led to remarkable cell death of organoids in a dose-dependent manner (Figure 2B, left), and significantly decreased the IC50 values of oxaliplatin (0.323 μM vs 5.661 μM) and 5-FU (5.493 μM vs 42.69 μM) in Organoid-1, of oxaliplatin (10.1 μM vs 33.72 μM) and 5-FU (49.52 μM vs 226.2 μM) in Organoid-2, and 5-FU (1.71 μM vs 273.7 μM) in Organoid-3, compared to respective controls (Figure 2B, right). *P < 0.05.As Organoid-3 was probably sensitive to oxaliplatin (IC50 6.506 μM), knockdown of hsa_circ_0020095 could not further decrease the IC50 value for oxaliplatin. We then adjusted the concentration ranges of 5-FU and oxaliplatin based on their IC50 values determined for each organoid line, and then used organoid lines 2 and 3 to verify the above observations. As expected, knockdown of hsa_circ_0020095 dramatically sensitized Organoid-2 to both 5-FU and oxaliplatin (Figure 3A), and Organoid-3 to 5-FU, while had no obvious effect on sensitivity of Organoid-3 to oxaliplatin (Figure 3B). **P < 0.01 compared to NC.

### Silencing of Hsa_circ_0020095 increases drug sensitivity possibly by modulating key pathophysiological processes

**FIGURE 4:**
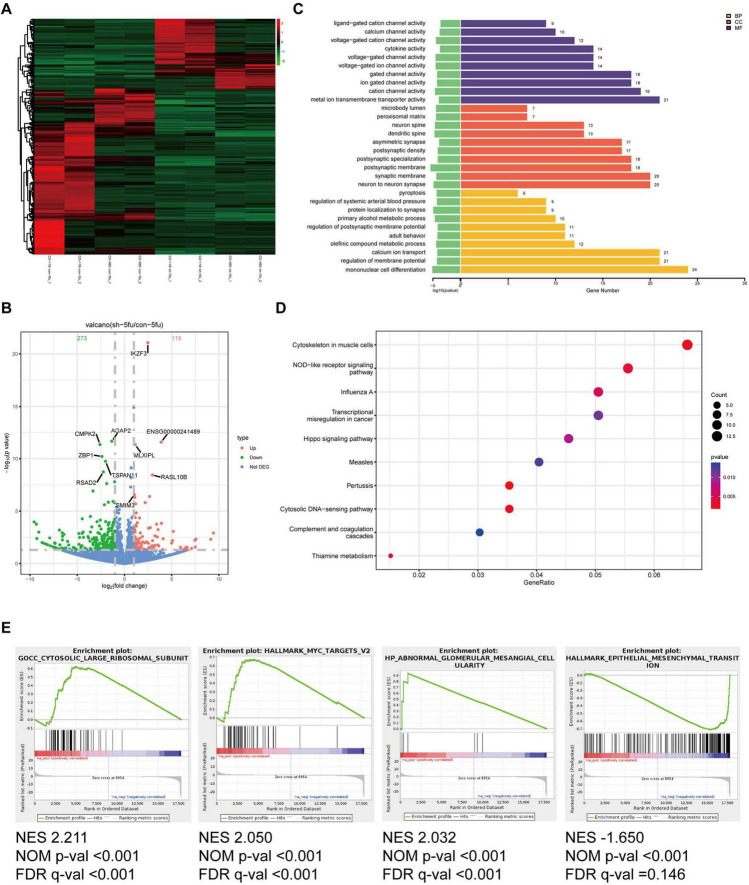
Potential roles of hsa_circ_0020095 in 5-fluorouracil (5-FU)-treated colorectal cancers (CRCs). Two organoid lines were transfected with hsa_circ_0020095 (KD) or control (NC) and treated with 5-FU, then underwent RNA-sequencing. Differentially-expressed genes (DEGs) were selected based on predefined criteria and shown on heat map and volcano plots **(A,B)**. Gene ontology (GO) **(C)**, Kyoto Encyclopedia of Genes and Genomes (KEGG) **(D)**, and Gene Set Enrichment Analysis (GSEA) **(E)** analyses were then conducted using these differentially-expressed genes (DEGs).

**FIGURE 5:**
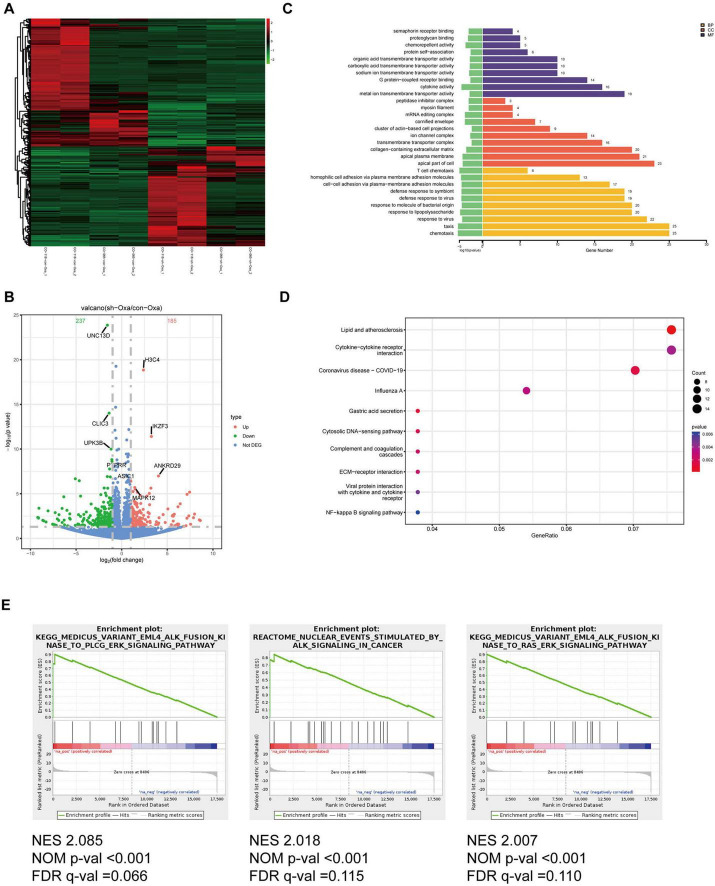
Potential roles of hsa_circ_0020095 in oxaliplatin-treated colorectal cancers (CRCs). Two organoid lines were transfected with hsa_circ_0020095 (KD) or control (NC) and treated with oxaliplatin, then underwent RNA-sequencing. Differentially-expressed genes (DEGs) were selected based on predefined criteria and shown on heat map and volcano plots **(A,B)**. Gene ontology (GO) **(C)**, Kyoto Encyclopedia of Genes and Genomes (KEGG) **(D)**, and Gene Set Enrichment Analysis (GSEA) **(E)** analyses were then conducted using these differentially-expressed genes (DEGs).

To explore the potential mechanism underlying the regulatory effects of hsa_circ_0020095 in drug resistance, we conducted transcriptomic sequencing using two organoid lines transfected with KD or NC and then treated with 5-FU or oxaliplatin (Figures 4A, 5A). We then selected DEGs based on predefined criteria as shown in volcano plots. There were 115 upregulated and 273 downregulated genes in organoids transfected with hsa_circ_0020095 and treated with 5-FU (the hsa_circ_0020095/5-FU group), while 185 upregulated and 237 downregulated genes in organoids transfected with hsa_circ_0020095 and treated with oxaliplatin (the hsa_circ_0020095/oxaliplatin group), compared to the controls (Figures 4B, 5B). We then performed GO and KEGG pathway analyses using these DEGs. GO analyses revealed that these DEGs were mainly enriched in biofunctions related to transmembrane transport and synapses for the hsa_circ_0020095/5-FU group (Figure 4C), and transmembrane transport, protein complex, receptor binding, and immune responses for the hsa_circ_0020095/oxaliplatin group (Figure 5C). KEGG analyses demonstrated that these DEGs were enriched in pathways such as those related to viral/bacterial infection and cell signaling transduction for the hsa_circ_0020095/5-FU group (Figure 4D), and viral/bacterial infection and ligand-receptor interaction for the hsa_circ_0020095/oxaliplatin group (Figure 5D). GSEA analyses suggested that knockdown of hsa_circ_0020095 and treatment with 5-FU were positively related to gene sets of GOCC_CYTOSOLIC_LARGE_RIBOSOMAL_SUBUNIT, HALL MARK_MYC_TARGETS_V2, and HP_ABNORMAL_GLO MERULAR_MESANGIAL_CELLULARITY, and negatively related to HALLMARK_EPITHELIAL_MESENCHYMAL_TRANSITION (Figure 4E), while knockdown of hsa_circ_0020095 and treatment with oxaliplatin were positively related to gene sets of KEGG_MEDICUS_VARIANT_EML4_ALK_FUSION_KINASE_ TO_PLCG_ERK_SIGNALING_PATHWAY, REACTOME_NU CLEAR_ EVENTS_ STIMULATED_ BY_ALK _SIGNALING_IN_ CANCER, and KEGG_MEDICUS_VARIANT_EML4_ALK_ FUSION_ KINASE_ TO_ RAS_ ERK_ SIGNALING_ PATHWAY (Figure 5E). Moreover, the expressions of ACTLB, CR2, CATSPERZ, WNGT5A, IL6R and TTYH2 in Oxaliplatin-treated CRCs were significantly upregulated by silencing of Hsa_circ_0020095 (Supplementary Figure 1). In contrast, knockdown of Hsa_circ_0020095 obvuously decreased the levels of PWWP38, KLF8, CXCL10, NLRP3, ZBP1 and IFNL2 in 5-FU/Oxaliplatin-treated CRCs (Supplementary Figure 2).

## Discussion

In the current study, we established five organoid lines from CRC patients and validated their resemblance to the parental tumors. The great advantage of organoids is that they are essentially the embodiment of the tumor in patients, resembling the source tumor in appearance and share many of the same molecular and genetic characteristics. Organoids often induce clonal evolution, which is a major contributor to tumor progression and drug resistance (14, 15). As PDOs conserve the heterogeneity of their source tumors, they are better models than the current cell lines for studies focusing on drug resistance of tumors.

Chemoresistance of CRC could lead to the failure of treatment and aggravation of patient outcome (16). Therefore, potential therapeutic targets are of special interest if they could be utilized to sensitize anticancer drugs. In the current study, we demonstrated that hsa_circ_0020095 could be such a therapeutic target, as knockdown of hsa_circ_0020095 enhanced the tumorcide capacity of 5-FU and oxaliplatin, both of which are cornerstone agents for anticancer therapies in CRC patients. In consideration of our previous observation that hsa_circ_0020095 may mediate cisplatin-resistance in colon cancers by modulating the miR-487a-3p/SOX9 axis (10), this research further supplemented the function of circRNAs in CRC, suggesting a promising target for cancer treatment. On the other hand, circRNAs are reported to be involved in CRC progression. For example, Hou et al. found METTL3-induced circ_0008345 can lead to the tumorigenesis of CRC via mediation of micRNA-182-5p/CYP1A2 axis (17); circPDIA3/miR-449a/XBP1 feedback loop could curb pyroptosis through suppressing palmitoylation of the GSDME-C domain to induce chemoresistance of CRC (18). Thus, more circRNAs other than hsa_circ_0020095 that are associated with the progression of CRC need to be further investigated.

Despite our novel discoveries, there are several limitations in this research as follows: (1) more downstream targets of hsa_circ_0020095 in CRC are needed to be further explored; (2) *in vivo* experiments were not included in this study; (3) the underlying mechanism should be further elaborated; (4) without blood as germline control. Hence, more investigations are essential in the coming future.

In summary, hsa_circ_0020095 may mediate drug-resistance of CRC to 5-FU and oxaliplatin by modulating key pathophysiological processes. Therefore, it could be potentially explored as a novel therapeutic target against CRC.

## Data Availability

The raw data supporting the conclusions of this article will be made available by the authors, without undue reservation.

## Supplementary material

Supplementary Figure 1 Upregulated expression profiles of mRNAs. 5-FU/oxaliplatin-treated CRCs were transfected with hsa_circ_0020095 (KD). The expressions of ACTLB, CR2, CATSPERZ, WNT5A, IL6R and TTYH2 in 5-FU/oxaliplatin-treated CRCs were detected using RT-qPCR. **P* < 0.05, ***P* < 0.01, ****P* < 0.001.

Supplementary Figure 2 Downregulated expression profiles of mRNAs. 5-FU/oxaliplatin-treated CRCs were transfected with hsa_circ_0020095 (KD). The expressions of PWWP38, KLF8, CXCL10, NLRP3, ZBP1 and IFNL2 in 5-FU/oxaliplatin-treated CRCs were detected using RT-qPCR. **P* < 0.05, ***P* < 0.01, ****P* < 0.001.
