# Supplementary figures and images for: Hsa_circ_0020095 modulates chemoresistance of CRC in a PDO model
Source: Front Med (Lausanne). 2025 May 27;12:1556611. doi: 10.3389/fmed.2025.1556611 (PMC12148843; doi:10.3389/fmed.2025.1556611)

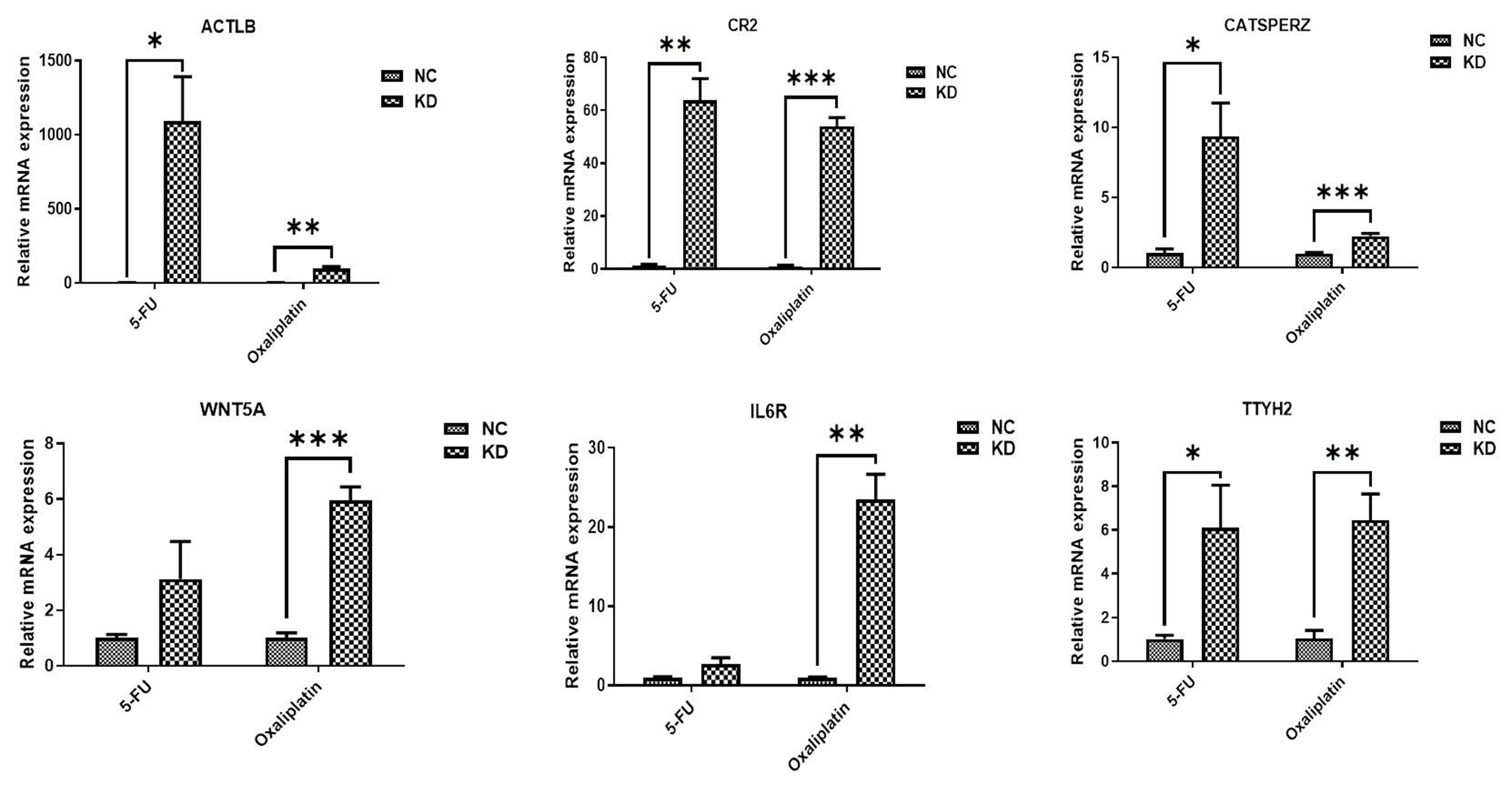

Supplement: Supplementary Figure 1 — Upregulated expression profiles of mRNAs. 5-FU/oxaliplatin-treated CRCs were transfected with hsa_circ_0020095 (KD). The expressions of ACTLB, CR2, CATSPERZ, WNT5A, IL6R and TTYH2 in 5-FU/oxaliplatin-treated CRCs were detected using RT-qPCR. *P < 0.05, **P < 0.01, ***P < 0.001. [file Image_1.JPEG]

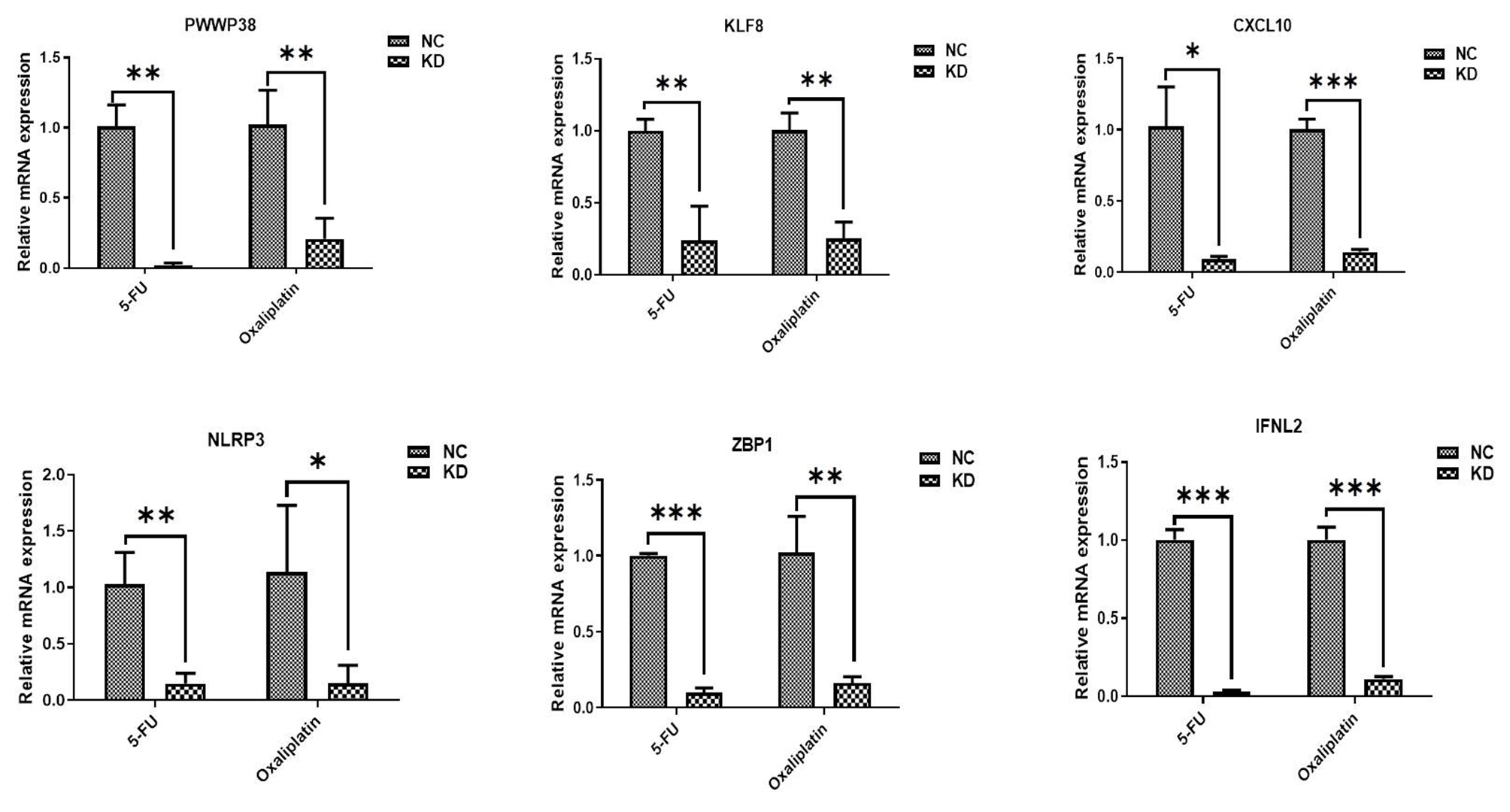

Supplement: Supplementary Figure 2 — Downregulated expression profiles of mRNAs. 5-FU/oxaliplatin-treated CRCs were transfected with hsa_circ_0020095 (KD). The expressions of PWWP38, KLF8, CXCL10, NLRP3, ZBP1 and IFNL2 in 5-FU/oxaliplatin-treated CRCs were detected using RT-qPCR. *P < 0.05, **P < 0.01, ***P < 0.001. [file Image_2.JPEG]
